# Is De-escalated Bisphosphonates Therapy a Suitable Alternative to Standard Dosing in Malignant Tumor Patients With Bone Metastases: A Systematic Review and Meta-Analysis

**DOI:** 10.3389/fonc.2019.00774

**Published:** 2019-08-14

**Authors:** Qiuhua Luo, Peng Men, Zhiyong Liu, Suodi Zhai, Mingyan Jiang

**Affiliations:** ^1^Department of Pharmacy, The First Affiliated Hospital of China Medical University, Shenyang, China; ^2^Department of Pharmacy, Peking University Third Hospital, Beijing, China; ^3^Liaoning Center for Drug and Device Evaluation and Monitoring, Shenyang, China

**Keywords:** bisphosphonates, bone metastases, de-escalated therapy, ONJ (osteonecrosis of the jaws), adverse effect

## Abstract

**Background:** Previous studies have preliminarily identified the non-inferior efficacy for reducing skeletal-related event (SRE) rates between de-escalated (Q12w) and standard (Q3-4w) bone-targeting agents therapy in malignant tumor patients with bone metastases. In this study, we aim to make further efforts to analyze whether the de-escalated bisphosphonates (BPs) strategy is a suitable option by comprehensively retrieving and synthesizing state-of-the-art evidence.

**Methods:** An extensive electronic search for randomized controlled trials (RCTs) comparing a BPs standard strategy with the de-escalated one in patients with bone metastases was performed up to June 2018. Outcomes of interest were general and found individual types of SRE, skeletal morbidity rate (SMR), bone pain, bone turnover biomarkers and adverse events (AEs). Continuous and dichotomous outcomes were summarized by the weighted mean difference (WMD) and risk ratio (RR), respectively, with 95% confidence intervals (CIs).

**Results:** A total of eight studies, representing six unique trials (involving 3114 patients), were included. Pooled results indicated comparable efficacy on general SRE (RR 0.99, 95% CI 0.87–1.12; *P* = 0.86; *I*^2^ = 0%) and SMR (WMD 0.00, 95% CI −0.02 −0.03; *P* = 0.81; *I*^2^ = 0%). However, the rate of surgery involving bones was significantly higher in de-escalated group than standard group (RR 1.92, 95% CI 1.17–3.15; *P* = 0.01; *I*^2^ = 0%) among individual types of SRE. Several trials also demonstrated increased levels of C-terminal or N-terminal telopeptide in de-escalated group. Meta-analyses for gastrointestinal disorders, dizziness and back pain showed significant reductions by 27% (RR 0.73, 95% CI 0.57–0.94; *P* = 0.01; *I*^2^ = 0%), 48% (RR 0.52 95% CI 0.32–0.86; *P* = 0.01; *I*^2^ = 0%), and 29% (RR 0.71, 0.51–0.99; *P* = 0.04; *I*^2^ = 0%), respectively, compared to the standard therapy.

**Conclusion:** For malignant tumor patients with bone metastases, a de-escalated BPs strategy is proved to have a better safety profile compared to standard dosing. Although the efficacy is generally comparable on SRE and SMR between the two dosing regimens, trials with long duration and large sample sizes are still warranted to make a solid judgment.

## Introduction

Bone metastases disease develops as a common clinical problem in malignant tumors. The pathophysiology of bone metastases causes a series of skeletal-related events (SREs), including severe bone pain, pathological fractures, hypercalcemia, and spinal cord compression (1–3). The associated complications caused by bone lesions, in consequence, increase the need for palliative radiotherapy or surgery to bone, which also leads to ongoing symptoms and deterioration of the quality of life (4).

The administration of bisphosphonates (BPs) has been confirmed to potently inhibit the osteoclast-mediated bone resorption, and therefore, delays the onset of skeletal complications (5–8). A recent systematic review of BPs for breast cancer treatment published in the Cochrane Library showed evidence that BPs reduce the risk of developing SREs, delay the median time to an SRE, and appear to reduce bone pain compared to placebo or none BPs therapy, for women with metastatic breast cancer and bone metastases (9).

Currently, monthly BPs therapy is recommended as standard care for patients diagnosed with bone metastases for at least 2 years, based on data derived from the studies of hypercalcaemia of malignancy (10–13). However, several studies have addressed the dosing interval and demonstrated that longer-interval dosing of BPs might maintains a comparable efficacy on reducing the risk of SREs along with reduced side effects. Particularly, it is noteworthy that the cumulative exposure of BPs is associated with significant toxicities, including osteonecrosis of the jaw (ONJ), renal dysfunctions, and gastrointestinal disorders, due to its relatively long half-life and preferential binding and accumulation in bone (14, 15). Their optimal dosing interval has accordingly come into question. On the other hand, as new anti-cancer agents continue to prolong the life expectancy of patients with malignant tumors, a suitable treatment schedule of BPs is required for a long-term medication to preferably increase the medication compliance (16).

A previous systematic review has explored de-escalated dosing of bone-targeting agents in patients with metastatic breast cancer (17). This systematic review performs a meta-analysis study of SRE, bone turnover biomarkers, and preliminarily identified the non-inferior efficacy between de-escalated (Q12w) and standard (Q3-4w) therapy. However, BP-specific results were not examined separately and individual types of SRE were not examined either. Some drug-related adverse reactions, including nausea, vomiting, abdominal pain, diarrhea, and constipation, that are commonly known to occur during treatment of bone-metastatic malignancies with BPs (18, 19), remain to be further evaluated through meta-analysis. It is therefore of interest to gain a more comprehensive understanding of the efficacy and safety profile of BPs administration in the comparison of different dosing regimens, among a broader population of cancer patients with bone metastases. Thus, we performed a state-of-the-art systematic review and meta-analysis, to further analyze whether the de-escalation strategy is a suitable option in patients with bone metastases from malignant tumors.

## Methods

The systematic review and meta-analysis were undertaken using a predetermined protocol and reported in accordance with the Preferred Reporting Items for Systematic Reviews and Meta-Analyses (PRISMA) statement (20).

### Literature Search Strategy

All relevant studies, describing randomized controlled trials (RCTs) of de-escalated (Q12w) vs. standard (Q3-4w) administration protocols for BPs treatment cycles were sought. An electronic literature search was performed from inception to July 2018 by searching the following databases: PubMed, Embase, and the Cochrane Library. The search strategy for PubMed was provided in Figure S1. Additional meeting abstracts were identified from meeting abstracts of American Society of Clinical Oncology, European Society for Medical Oncology, American Association for Cancer Research n and European Association for Cancer Research via Embase. ClinicalTrials.gov was further searched to ensure the identification of published and unpublished RCTs. We also manually searched the reference lists of relevant studies.

### Study Selection and Data Extraction

The eligibility criteria for the systematic review was in accordance with the PICOS (participants, interventions, comparators, outcomes, and study design) approach. RCTs meeting the following criteria were considered for inclusion:

- Participants: patients with malignant tumors who had at least one site of bone involvement.
- Interventions: BPs administrated iv Q12w as a de-escalated arm.
- Comparators: BPs administrated iv Q12w vs. Q3-4w.
- Outcomes: SRE-related outcomes (general SRE, individual types of SRE and time-to-first on-study SRE), skeletal morbidity rate (SMR), bone pain, changes of bone turnover biomarkers and adverse event (AE).

The screening of titles, abstracts, and full-text references was performed by two reviewers, independently, to identify a set of potentially relevant citations. Data extraction was collected and arranged by researchers using a collection form. Publication sources, intervention details, patient inclusion criteria, and demographics, as well as outcome measures mentioned earlier, were extracted. When detailed full-texts were not available, the relevant contents from abstracts were used for data extraction (but not assessed for risk of bias). Corresponding authors were contacted for data not available within studies, or when outcomes were presented in an unsuitable format for data synthesis.

### Risk of Bias

The quality of individual studies was assessed by using the Cochrane Collaboration's risk of bias tool according to five domains as follows: random sequence generation (selection bias); allocation concealment (selection bias); blinding (performance bias and detection bias); incomplete outcome data (attrition bias); and selective reporting (reporting bias). Any discrepancy in the quality of RCTs was resolved by discussions among two reviewers, or by the assistance of a third researcher if necessary.

### Data Analysis

Meta-analysis was performed by Review Manager 5.2 software (RevMan, Cochrane, London, UK). The continuous outcome was summarized by WMD, while the dichotomous outcome was summarized by RR with corresponding 95% confidence intervals (CIs). Statistical homogeneity among RCTs was calculated by using the chi-square test and *I*^2^*-*value. The outcome data were synthesized by random-effect models (*I*^2^ > 50%) or fixed-effect models (*I*^2^ ≤ 50%), respectively, depending on the amount of heterogeneity observed. Forest plots and study-level effect estimates were designed to present various research indicators. If the extracted data was not sufficient for a quantitative meta-analysis, a narrative approach was conducted to summarize the study-specific results.

## Results

### Selection of Studies

**Figure 1:**
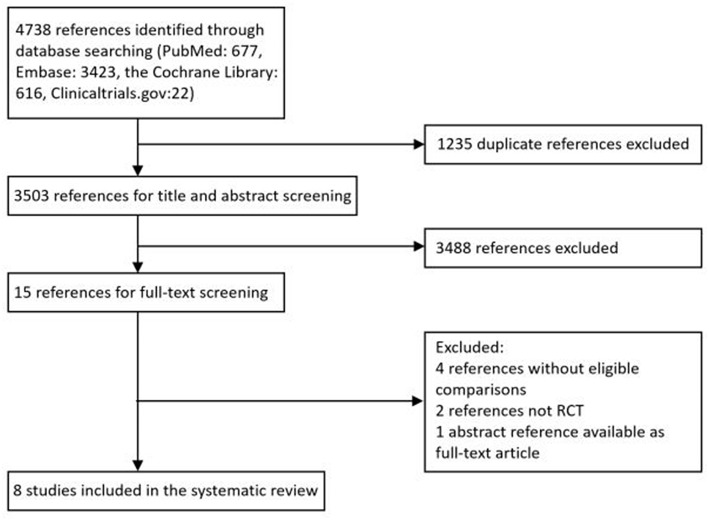
Flow chart of the study selection process.

A total of 4,738 citations were retrieved through electronic search from Pubmed, Embase databases and the Cochrane library, from which 1,235 duplicated records were excluded and 3,503 potentially eligible reports were identified by reviewing study titles and abstracts. Based on the inclusion criteria established for the present study, an additional record was obtained from *ClinicalTrial.gov*. After the full-text screening, a total of eight RCTs consisting of 3114 patients were included, among which one study was closed early as a consequence of slow patient accrual. Figure 1 provides an overview of the process of study selection.

### Study Characteristics

Characteristics of included studies were described in Table 1, including study design, interventions, sample size, patient inclusion criteria, outcomes measure, and study duration. One study was published in 2012 (24), three were in 2013 (14, 22, 26), one was in 2014 (21), and the other two were in 2017 (23, 25). Two studies were of different design in dose administration compared to the other de-escalation studies, as each zoledronate dose was adjusted for calculated creatinine clearance using actual body weight (23) or the dosing interval was based on bone turnover biomarker levels of the individual patient (24). One study was from Phase I clinical trial results (ClinicalTrials.gov Identifier: NCT00424983) (27), which had never been published in a peer-reviewed journal. Because of the limited information given in the trial registry, the detailed data were retrieved from the trial's sponsor (Novartis). One study published updated data from the included studies (27). The BISMARK study closed before reaching the primary endpoint, and data was obtained from updated study reports from the American Society of Clinical Oncology (ASCO) meetings (24). The BPs evaluated within studies were either pamidronate or zoledronate, and the study duration varied between 1 and 2 years.

**Table 1:** Overview of characteristics of included studies.

**Study**	**Study design**	**Interventions (*****n*****)**		**Sample size (n)**	**Mean age (years)**	**Patient inclusion criteria**	**Outcomes measure**	**Duration**
		**De-escalated**	**Standard**					
Addison et al. (21)	Pilot, randomized, non-inferiority trial (data from the pilot, feasibility randomized trial)	90 mg pamidronate i.v. Q12w (17); concomitant VD_3_ (800–1,000 IUs/day) and Ca (1,200–1,500 mg/day)	90 mg pamidronate i.v. Q3-4w (13); concomitant VD_3_ (800-1000 IUs/day) and calcium (1,200–1,500 mg/day)	30	NR	MBC; baseilne serum CTx < 600 ng/L; ≥ 3 months of prior anti-bone resorption therapy	CTx, BAP, BPI, FACT-BP	1 year
Amadori et al. (14) (ZOOM)	Multicenter, prospective, randomized, open-label, non-inferiority trial	4 mg zoledronate i.v. Q12w (209); supplementary calcium (500 mg/day) and VD (400–500 IUs/day)	4 mg zoledronate i.v. Q4w (216); supplementary calcium (500 mg/day) and VD (400–500 IUs/day)	425	Q4w: 59.8 (Median) Q12w: 60.4 (Median)	Stage IV MBC; 12-15 months of prior zoledronate use	SMR, the incidence of SRE/year, time-to-first SRE, bone pain, NTx	1 year
Amir et al. (22) (REFORM)	Randomized feasibility study	90 mg pamidronate i.v. Q12w (19)	90 mg pamidronate i.v. Q3-4w (19)	38	Q3-4w: 55 (Median) Q12w: 60 (Median)	MBC; baseline serum CTx < 600 ng/L; ≥ 3 months of prior BPs use	CTx, BAP, FACT-BP, BPI, SRE	1 year
Himelstein et al. (23)	Randomized, open-label clinical trial	Each zoledronate dose was adjusted for calculated creatinine clearance using actual body weight, i.v., Q12w (911); 500 mg calcium and 400–800 IUs VD/day	Each zoledronate dose was adjusted for calculated creatinine clearance using actual body weight, i.v., Q4w (911); 500 mg calcium and 400–800 IUs VD/day	1822	Q3-4w: 65 (Median) Q12w: 66 (Median)	MBC, MPC, MM; ECOG score of 0 to 12, Cr ≥ 20 mL/min, serum calcium level between 8.0 mg/dL to 11.6 mg/dL; No prior BPs	SRE rate, BPI, ECOG, the incidence of ONJ and kidney dysfunction, SMR, CTx	2 year
Coleman et al. (24)(BISMARK)	Open-label, randomized trial	M-ZOL (Q15-16w; Q8-9w or Q3-4w)	S-ZOL (Q3-4w)	289	NR	MBC; No prior BPs	SRE, SMR	2 year
Hortobagyi et al. (25) (OPTIMIZE-2)	Prospective, randomized, double-blind, multicenter clinical trial	4 mg zoledronate i.v. Q12w (203)	4 mg zoledronate i.v. Q4w (200)	416	Q3-4w: 59.2 (Mean) Q12w: 58.6 (Mean) placebo: 60.8 (Mean)	MBC; 10–15 months of prior zoledronate and/or pamidronate use	SRE rate, time-to-first SRE, SMR	1 year
Kuchuk et al. (26)	Updated data from REFORM study	90 mg pamidronate i.v. Q12w (19)	90 mg pamidronate i.v. Q3-4w (19)	38	NR	MBC; baseilne serum CTx < 600 ng/L; ≥3 months of prior BPs use	Correlation between pain scores and CTx, FACT-BP scores	1 year
ClinicalTrials.gov Identifier: NCT00424983 (27)	Randomized, open-label, multi-center, comparative 2-arm trial	4 mg zoledronate i.v. Q12w (9)	4 mg zoledronate i.v. Q4w (9)	18	Q3-4w: 62.0 (Median) Q12w: 60.0 (Median)	MBC, MM; 9 to 12 infusion of zoledronate during the previous year	SRE, SMR, AUC_0−24h_, CLcr	1 year

*MBC, metastatic breast cancer; MPC, metastatic prostate cancer; MM, multiple myeloma; SRE, skeletal-related events; SMR, skeletal morbidity rate; CTx, serum C-terminal telopeptide; NTx, urinary N-terminal telopeptide; BAP, alkaline phosphatase; CLcr, creatine clearance; FACT-BP, Functional Assessment of Cancer Therapy Bone Pain; BPI, Brief Pain Inventory; ECOG, Eastern Cooperative Oncology Group; NR, not reported*.

### Patient Characteristics

Patients diagnosed with metastatic breast cancer (MBC), metastatic prostate cancer (MPC) or multiple myeloma (MM) were randomly assigned to a de-escalated dosing or standard dosing of BPs. Three used additional study entry criteria based on low baseline serum C-terminal telopeptide (CTx, < 600 ng/L) (21, 23, 26). One study included Eastern Cooperative Oncology Group (ECOG) performance status score of 0 to 2, calculated creatinine clearance of ≥ 30 mL/min and serum calcium level between 8.0 mg/dL and 11.6 mg/dL as inclusion criteria (23). The prior treatment of BPs used before enrollment varied from 0 to 15 months.

### Risk of Bias Assessment

**Figure 2:**
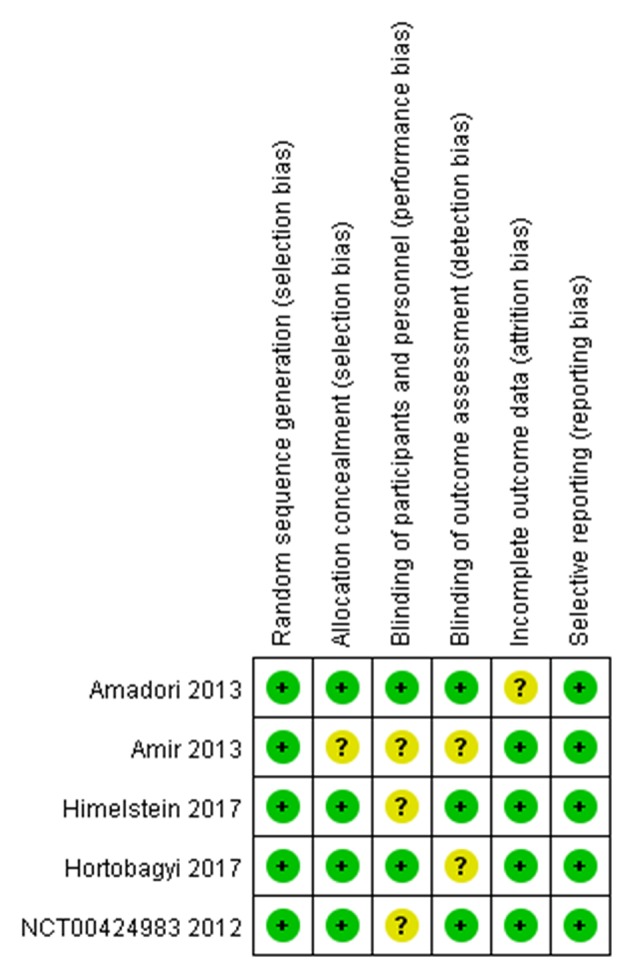
Risk of bias table with the five studies included in the systematic review.

The assessment of the risk of bias for individual studies was summarized in Figure 2. Five full-text articles were available for risk of bias assessment (14, 22, 23, 25). Two studies were excluded from bias assessment as the study used the same randomized trial data as one of the included studies (21, 26). Four studies were explicit about each allocation concealment and blinding of the outcome assessment (14, 22, 23, 25).

### SRE-Related Outcomes

#### General SRE

**Figure 3:**
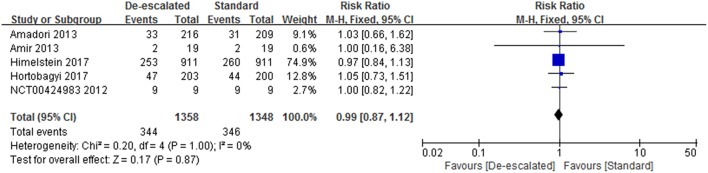
Meta-analysis result of SREs.

Studies reported a number of SRE-related outcomes including the SRE rate, SMR, and time-to-first on-study SRE. Five included studies (2,703 patients) investigated the effect of different dosing interval of BPs on the SRE rate (14, 22, 23, 25, 27). A pooled RR of 0.99 (95% CI 0.87–1.12; *P* = 0.86) indicated that there was no evidence of a significant difference in SRE rate between de-escalated and standard arms (Figure 3). No heterogeneity was observed amongst the studies (*I*^2^ = 0%). The BISMARK study closed before reaching its primary endpoint, however, it showed no statistically significant difference in the occurrence of SREs between the different intervention groups (24). In addition, two studies reported data of the time of the onset of first SRE. The OPTIMIZE-2 study showed time-to-first SRE between treatment groups was not statistically different (hazard ratio, 1.06; 95% CI, 0.70–1.60; *P* = 0.79) (25). While the ZOOM study presented that the median time to first on-study SRE could not be calculated because of the very low event rate (14).

#### Individual Type of SRE

**Figure 4:**
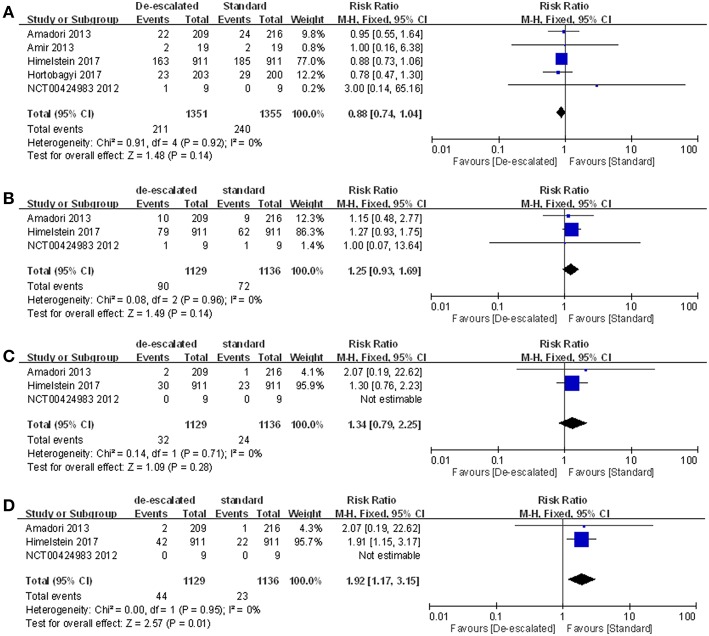
Meta-analysis results of individual types of SRE: **(A)** radiation to bone, **(B)** clinical fractures, **(C)** spinal cord compression, and **(D)** surgery involving bones.

Studies reported a number of individual types of SRE, including radiation to bone, clinical fractures, spinal cord compression, and surgery involving bone. The most frequently recorded individual type of SRE in both treatment groups was radiation to bone in de-escalated vs. standard group (15.62 vs. 17.71%), followed by pathological fractures (7.97 vs. 6.43%), surgery involving bone (3.90 vs. 2.02%) and spinal cord compression (2.83 vs. 2.11%) (Table S1). A summary RR of 0.88 (95% CI 0.74–1.04; *P* = 0.92; *I*^2^ = 0%) (14, 22, 23, 25, 27), 1.25 (95% CI 0.93–1.69; *P* = 0.14*; I*^2^ = 0%) and 1.34 (95% CI 0.79–2.25; *P* = 1.34*; I*^2^ = 0%) indicated there was no statistically significant differences between the arms, with respect to radiation to bone, clinical fractures and spinal cord compression, respectively (14, 23, 25). However, comparison in the aspect of surgery involving bone produced a pooled RR of 1.92 (95% CI 1.17–3.15; *P* = 0.01*; I*^2^ = 0%; Figure 4) (14, 23, 25).

### Skeletal Morbidity Rates (SMR)

**Figure 5:**
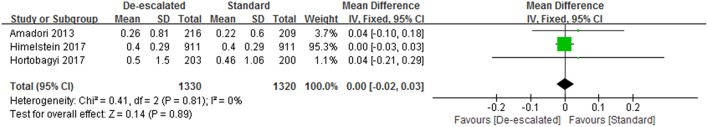
Meta-analysis result of SMR.

Three studies reported SMR data involved with zoledronate and manifested de-escalated therapy with BPs did not appear to affect an overall SMR (WMD 0.00, 95% CI −0.02 – 0.03; *P* = 0.81; *I*^2^ = 0%; Figure 5) (14, 23, 25). Besides, the BISMARK study, designed by a fundamentally different form as mentioned before, reported SMR of 0.52 (90% CI 0.47–0.58) and 0.72 (90% CI 0.67–0.77) for the standard and bone-biomarker-directed therapy, respectively (24).

### Bone Pain

Four studies reported data of bone pain, while differed in the employed assessment tool for pain response. Thus, a meta-analysis was not carried out. The ZOOM study, relying on the Verbal Rating Score (VRS), detected no relevant differences between groups for bone pain at the end of study or analgesic use during follow-up. The median pain at rest and on movement scores were <4 at all points in both groups. The REFORM study and Addison et al. demonstrated that the pain scores remained generally stable over time in both the standard and the de-escalated groups, according to validated Brief Pain Inventory (BPI) and Functional Assessment of Cancer Therapy-Bone Pain (FACT-BP). There were no statistically significant differences between groups in cumulative pain scores as measured by BPI (*P* = 0.21) or by FACT-BP (*P* = 0.59) (21, 22). Himelstein et al. also used ECOG performance status to evaluate bone pain data, showing the score of 0.025 for every 4 weeks and 0.024 for every 12 weeks. Similarly, the result showed no statistically significant differences at each time point (*P* = 0.64), as well as mean worst pain within the past 24 h (0.022 vs. 0.021, *P* = 0.96), mean current pain (0.016 vs. 0.018, *P* = 0.82), composite pain (0.021 vs. 0.022, *P* = 0.88), mean relief from pain with treatments or medications (0.009 vs. 0.016, *P* = 0.59), and mean interference score (0.023 vs. 0.019, *P* = *0.68*) comparing the de-escalated and standard group (*P* > 0.001) (23).

### AEs

**Figure 6:**
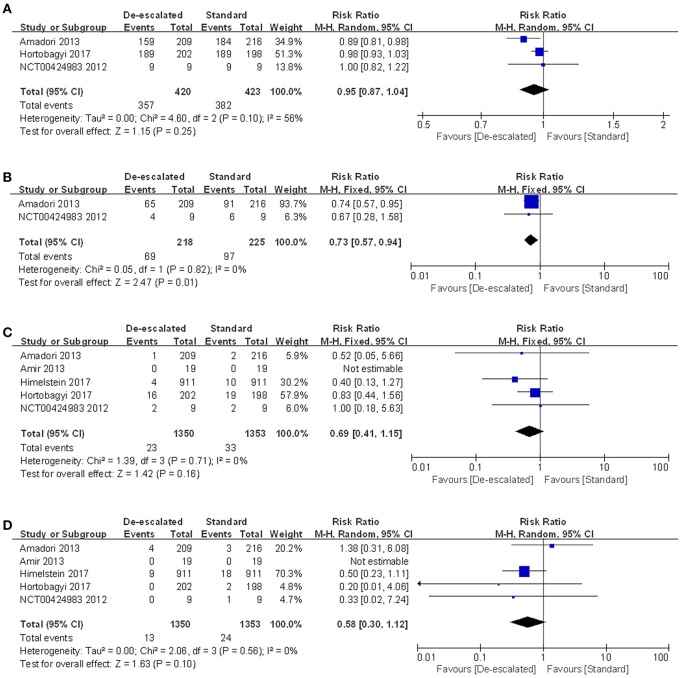
Meta-analysis results of safety outcomes: **(A)** AEs, **(B)** gastrointestinal disorders, **(C)** renal dysfunction and **(D)** ONJ.

AEs occurred more commonly in the standard group than in the de-escalated group, which might be driven by higher incidence of renal dysfunction, ONJ and gastrointestinal disorders (including nausea, vomiting, abdominal pain, diarrhea, and/or constipation) with more frequent medication. A series of specific toxicities were described in detail by treatment comparison in the form of forest plots (Figure 6 and Figures S2–S5). Data were available from five studies reporting on-study AEs for meta-analysis (Figure 6A). Three studies reported the number of patients with at least one AE, and the result of the meta-analysis showed no evidence of a difference between the arms (RR 0.95, 95% CI 0.87–1.04; *P* = 0.25) with the random-effect model (*I*^2^ = 56%) (14, 25, 27). For on-study renal dysfunction, comparison of de-escalated and standard treatment groups using meta-analysis produced a pooled RR of 0.69 (95% CI 0.41–1.15; *P* = 0.16*; I*^2^ = 0%; Figure 6C) (14, 22, 23, 26, 27). For on-study ONJ, comparison of treatment groups produced a pooled RR of 0.56 (95% CI 0.29–1.08; *P* = 0.10; Figure 6D). No heterogeneity was detected (*I*^2^ = 0%) (14, 22, 23, 26, 27). Neither renal dysfunction nor ONJ was observed in the REFORM study, probably due to the small number of recruitment. Notably, the de-escalated therapy of BPs reduced the incidence of gastrointestinal disorders by 27% compared to the standard therapy (RR 0.73, 95% CI 0.57–0.94; *P* = 0.01; *I*^2^ = 0%; Figure 6B) (14, 27). The gastrointestinal reactions were further refined into nausea, vomiting, decreased appetite, diarrhea, constipation, and abnominal pain. The numbers of patients who experienced these symptoms were processed for meta-analyses. The results showed a summary RR of 0.84 (95% CI 0.65–1.09; *P* = 0.19; *I*^2^ = 0%) (14, 25, 27), 0.85 (95% CI 0.60–1.20; *P* = 0.35; *I*^2^ = 9%) (14, 25, 27), 0.92 (95% CI 0.58–1.46; *P* = *0.72*; *I*^2^ = 26%) (14, 27), 0.83 (95% CI 0.59–1.17; *P* = 0.29; *I*^2^ = 0%) (14, 25, 27), 0.78 (95% CI 0.55–1.11; *P* = 0.16; *I*^2^ = 0%) (14, 25, 27) and 0.96 (95% CI 0.48–1.93; *P* = 0.92; *I*^2^ = *29%*) (14, 27), respectively, corresponding to the above mentioned symptoms.

Other AEs were also collected such as hyocalcemia, anemia, arthralgia, pain in extremity, headache, edema peripheral, cough, neutropenia, musculoskeletal pain, dyspnoea, grade 3–4 AEs, dizziness and back pain. Based on the extracted data, we conducted meta-analyses with corresponding summary RR results of 0.91 (95% CI 0.80–1.03; *P* = 0.14; *I*^2^ = 0%) (23, 27), 0.98 (95% CI 0.68–1.42; *P* = 0.91; *I*^2^ = 38%) (22, 25, 27), 0.82 (95% CI 0.62–1.08; *P* = 0.15; *I*^2^ = 0%) (14, 25, 27), 0.88 (95% CI 0.64–1.21; *P* = 0.44; *I*^2^ = 0%) (14, 25, 27), 1.00 (95% CI 0.69–1.43; *P* = 0.98; *I*^2^ = 0%) (14, 25, 27), 0.93 (95% CI 0.62–1.39; *P* = 0.73; *I*^2^ = 0%) (14, 25, 27), 1.23 (95% CI 0.80–1.87; *P* = 0.34; *I*^2^ = 0%) (14, 25, 27), 0.55 (95% CI 0.28–1.10; *P* = 0.09; *I*^2^ = 1%) (14, 27), 0.95 (95% CI 0.81–1.10; *P* = 0.50; *I*^2^ = 0%), 0.80 (95% CI 0.54–1.20; *P* = 0.29; *I*^2^ = 0%) (14, 25) and 0.89 (95% CI 0.57–1.40; *P* = 0.64; *I*^2^ = *0%*) (14, 27). For dizziness and back pain, de-escalated therapy could induce significant decreases (RR 0.52 95% CI 0.32–0.86; *P* = 0.01; *I*^2^ = 70% and RR 0.69 95% CI 0.49–0.96; *P* = 0.03; *I*^2^ = 0%) (25, 27).

In addition, the study by Himelstein et al. in which all patients received daily supplementation with calcium and vitamin D, demonstrated that the rate of hypocalcemia driven by zoledronate was 32.7% for the de-escalated group and 35.3% for the standard group with no statistical difference (*P* = 0.25), respectively (23).

### Biomarkers of Bone Turnover

The change in biomarker levels on study was assessed, given that they have been used in clinical trials as a surrogate marker of bone metastases control and hence BPs efficacy (28–30). Four studies reported biomarkers of bone turnover in the form of CTx and urinary N-terminal telopeptide (NTx). Addison et al. reported that, compared to the standard group, patients in the de-escalated group had statistically significantly greater increases in CTx levels (median of 131 vs. 17, *P* = 0.034) and no statistically significant change in NTx (median of 45 vs. 43, *P* = 0.54) (21). The ZOOM study assessed median percentage change in NTx concentration from baseline and indicated it was significantly higher in the de-escalated group vs. the standard group at 6 months (12.2 vs. −2.3%; *P* = 0.011), 9 months (10.6 vs. −2.2%; *P* = 0.047), and 12 months (12.2 vs. 0.0%; *P* = 0.047) (14). The REFORM study showed a consistent and maintained increase in absolute CTx levels in women receiving de-escalated therapy, which met the prespecified cutoff for clinical significance (*P* = 0.096) (22). Himelstein et al. made a descriptive evaluation of CTx measurement results (23). The observed CTx levels were higher at each time point among patients receiving the de-escalated zoledronate but involved no statistical test. Given the different scales in the reporting of outcomes, a meta-analysis was not carried out.

## Discussion

The rational dosing interval of BPs has gradually attracted attention especially to oncologists and patients with bone metastases, due to the increased toxicity caused by a long-term medication (31, 32). Along with the advancing clinical studies being designed to explore its optimal dosing frequency, it is of the utmost importance to gain a better understanding of the fact that whether a less intensive dosing schedule of BPs therapy might provide the comparable efficacy as the standard one while reducing side effects (33).

First of all, as the most commonly used efficacy evaluated outcome, SRE-related data was available in a total of six studies, in terms of SRE rate, SMR and time-to-first SRE. The meta-analysis result of the number of patients having an on-study SRE showed evidence that a longer-interval BPs regimen (Q12w) was similar to a standard schedule (Q3-4w) toward patients with bone metastases. Meanwhile, there was no evidence of a difference in SMR between the de-escalated and standard groups, with an inconclusive CI and summary estimate near the null (RR 0.00, 95% CI −0.02 – 0.03; *P* = 0.89; *I*^2^ = 0%). Two articles reported time-to-first SRE and showed no significant statistical difference between the two treatments (14, 25). Unfortunately, one of the articles only stated descriptive results. There was no sufficient, consistent data for a meta-analysis to be carried out. Furthermore, individual types of SREs were analyzed, including radiation to bone, clinical fractures, spinal cord compression and surgery involving bone. It is noteworthy that the de-escalated regimen led to a significantly increased incidence of surgery involving bone compared with the standard care, despite the comparable result of the general SRE rate. Consequently, we consider this to be a point of concern in evaluating the efficacy of both treatments.

Secondly, following a thorough review of toxicity, there were new safety findings according to the meta-analysis results. The proportion of patients who experienced at least one AE revealed a slightly decreased tendency in the longer-interval dosing regimen, although this was not statistically significant. Nevertheless, de-escalated regimen did provide an overall benefit by reducing the risk of gastrointestinal disorders, back pain and dizziness by 27, 31, and 48%, respectively, comparing to the standard therapy (Figure 6B, Figures S3, S4). Besides, the de-escalated dosing of BPs slightly depressed the risk of acute-phase reactions (such as fatigue and nausea), although there was no statistical significance (Figure S1).

For on-study renal dysfunction and ONJ, different dosing frequencies with BPs did not significantly affect the occurrence rate, although data from individual studies would suggest that standard dosing was associated with an increased rate (23, 25). However, it needs to be pointed out that as the most serious type of AEs, ONJ occurs after cumulative dosing of BPs, and it is therefore likely that some included studies with limited follow-up period would not allow for the detection of such event (34–36). Longer-term studies might be necessary to show any benefit from de-escalated dosing on the occurrence of ONJ.

Furthermore, circulating telopeptide levels have been used as endpoints to determine drug efficacy and an indicator for the relationship with SRE risk as well (37, 38). The measurement index for turnover biomarkers varied among studies, by using different indicators such as NTx and CTx, hence data extracted was not consistent enough for pooled analysis. In general, the observed levels of bone turnover biomarkers were higher at each time point among patients receiving BPs in de-escalated groups. In the ZOOM study and the Addison study, there is even a significant difference in median percentage change from baseline in NTx and CTx concentration in the 4-week group vs. the 12-week group (14, 21). The recovery of bone turnover is probably a result of the rapid removal of stored BPs through increased osteoclast activity at sites of active bone lesions (16, 39). Despite this, given that an increasing incidence of surgery involving bone was directly related to the de-escalated regimen, the significance of certain biomarkers should be highly stressed in the future work. These findings raise questions around the long-term efficacy of de-escalation of bone-targeted therapy as well. On the other hand, the available data of recent work highlighted the need for completion and analysis of larger definitive trials to assess the roles of biomarkers as predictors of SRE risk.

This review has a few limitations that need to be addressed. First, there was clinical and methodological heterogeneity among studies in terms of duration of studies and different schedules of prior BPs usage. Secondly, one study terminated early due to challenges with enrollment, achieving only 289 of the targeted 1,400 (24); as such, it was unclear whether study findings might have changed with additional recruitment. Thirdly, only trials among MBC, MPC, or MM patients were available to be analyzed. Therefore, further studies are still warranted to make a final conclusion as to whether the results can be generalized to other types of cancers. Finally, the study did not address any survival data, risks or benefits of administering BPs for more than 2 years. As tumor bone metastases are equivalent to chronic diseases that require long-term treatment of BPs, corresponding follow-up data of more than 2 years should be necessary. Despite these limitations, it is of interest to note that there were consistent patterns across all trials, all of which were multi-center studies and most of which involved multi-ethnic patients. These findings confirmed that a longer-interval of BPs was similar to the standard therapy in reducing the risk of developing SREs. What's more, the de-escalated regimen was proven for the first time to provide an overall safety benefit by reducing the risk of gastrointestinal disorders and significantly relieving the phenomenon of back pain.

## Conclusions

For malignant tumor patients with bone metastases, a de-escalated BPs strategy is proven to have a better safety profile compared to standard dosing. Although the efficacy is generally comparable on SRE and SMR between the two dosing regimens, trials with long durations and large sample sizes are still warranted to make a solid judgment.

## Data Availability

All datasets generated for this study are included in the manuscript and/or the Supplementary Files.

## Author Contributions

QL and PM conceived the study and collected data. ZL analyzed the data. SZ and MJ verified the data. QL, PM, and ZL prepared and edited the manuscript. All authors contributed to the paper and perspectives, and agreed with its final content.

### Conflict of Interest Statement

The authors declare that the research was conducted in the absence of any commercial or financial relationships that could be construed as a potential conflict of interest.
