# Supplementary material for: Is De-escalated Bisphosphonates Therapy a Suitable Alternative to Standard Dosing in Malignant Tumor Patients With Bone Metastases: A Systematic Review and Meta-Analysis
Source: Front Oncol. 2019 Aug 14;9:774. doi: 10.3389/fonc.2019.00774 (PMC6702312; doi:10.3389/fonc.2019.00774)
Supplement: Supplementary file 1 [file Table_1.DOCX]

| Database: PubMed | |
| --- | --- |
| ***Line #*** | ***Search strategy*** |
| 1 | Neoplasms[MeSH Terms] OR cancer*[tiab] OR carcinoma*[tiab] OR neoplasm*[tiab] OR tumor*[tiab] |
| 2 | Bone and Bones[MeSH Terms] OR bone[tiab] OR bones[tiab] |
| 3 | Neoplasm Metastasis[MeSH Terms] OR metastases[tiab] OR metastasis[tiab] OR metastatic[tiab] OR micrometastases[tiab] OR micro-metastases[tiab] OR micrometastasis[tiab] OR micro-metastasis[tiab] OR recurrence*[tiab] OR recrudescence*[tiab] OR recurrent[tiab] OR secondary[tiab] OR spread*[tiab] |
| 4 | #1 AND #2 AND #3 |
| 5 | Diphosphonates[MeSH Terms] OR "diphosphonates"[tiab] OR "bisphosphonates"[tiab] |
| 6 | Search alendronate[tiab] OR etidronate[tiab] OR zoledronic acid[tiab] OR ibandronate[tiab] OR risedronate[tiab] OR pamidronate[tiab] OR tiludronate[tiab] OR olpadronate[tiab] OR neridronate[tiab] OR Clodronate[tiab] OR cimadronate[tiab] |
| 7 | Search (#5 OR #6) |
| 8 | Search randomized controlled trial[pt] |
| 9 | Search (random*[tiab] or placebo*[tiab] or single blind*[tiab] or double blind*[tiab] or triple blind*[tiab]) |
| 10 | Search (#8 OR #9) |
| 11 | #4 AND #7 AND #10 |

**Figure S 1 Summary of electronic literature search of PubMed**


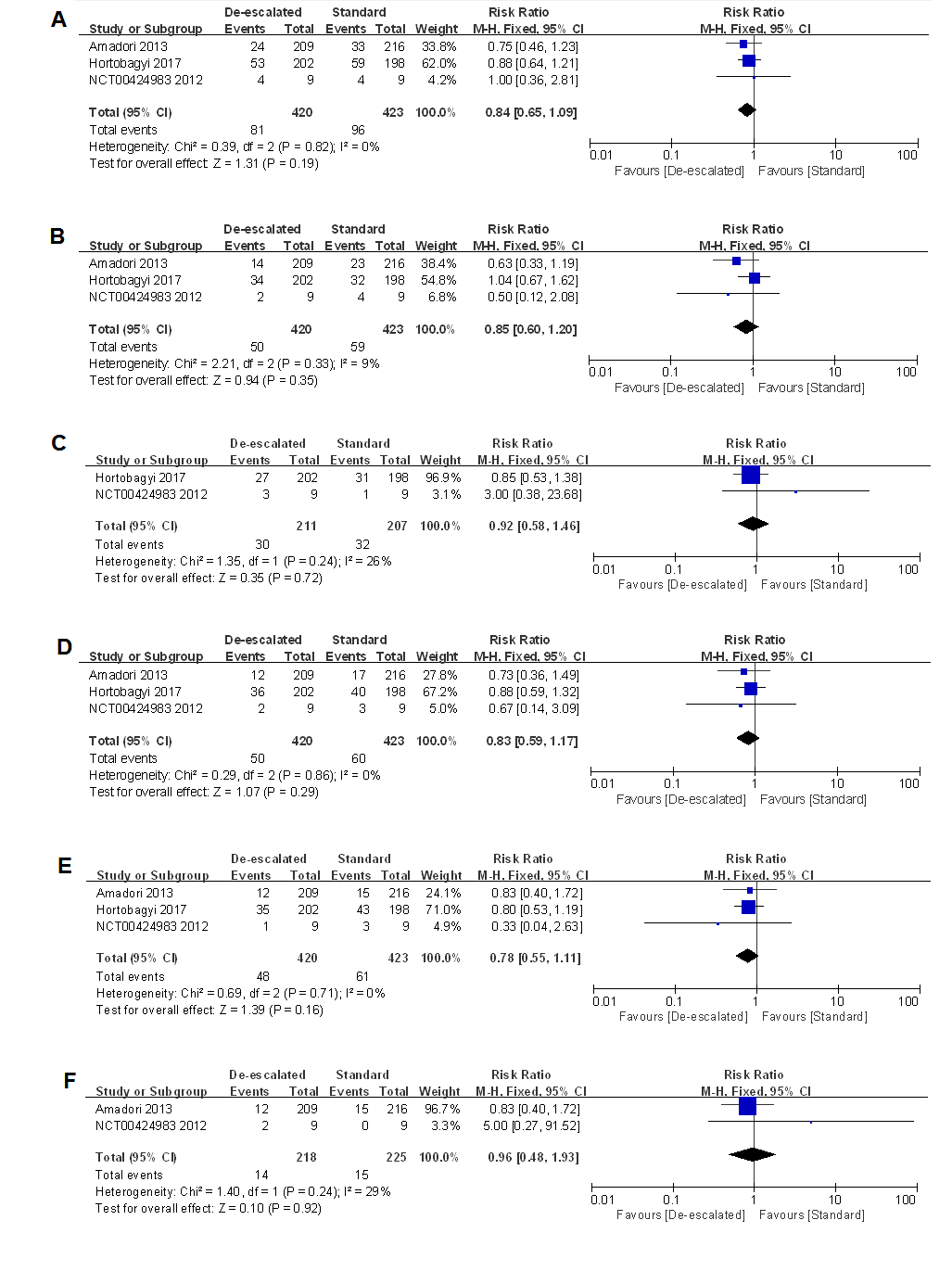


**Figure S 2 On-study (A) nausea, (B) vomiting, (C) decreased appetite, (D) diarrhea, (E) constipation and (F) abnominal pain**


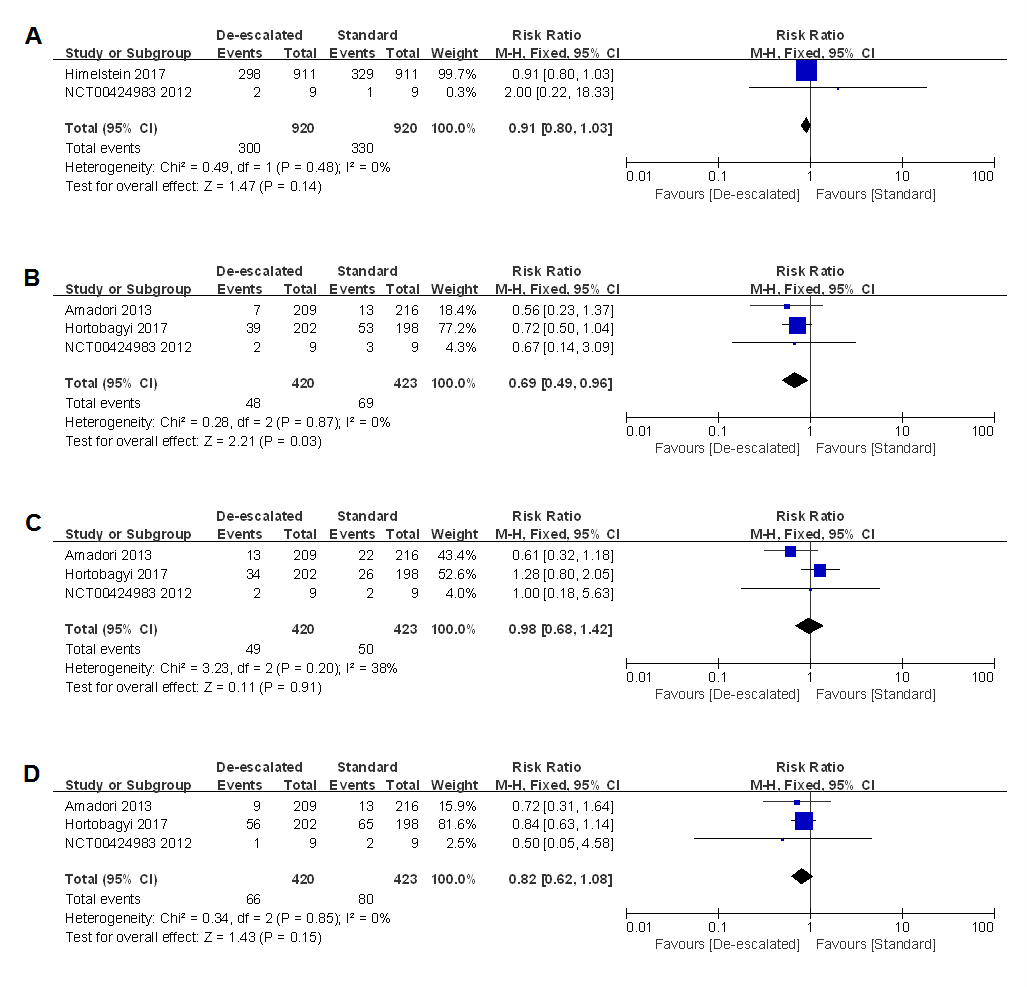


**Figure S 3 On-study (A) hyocalcemia, (B) back pain, (C) anemia and (D) arthralgia**


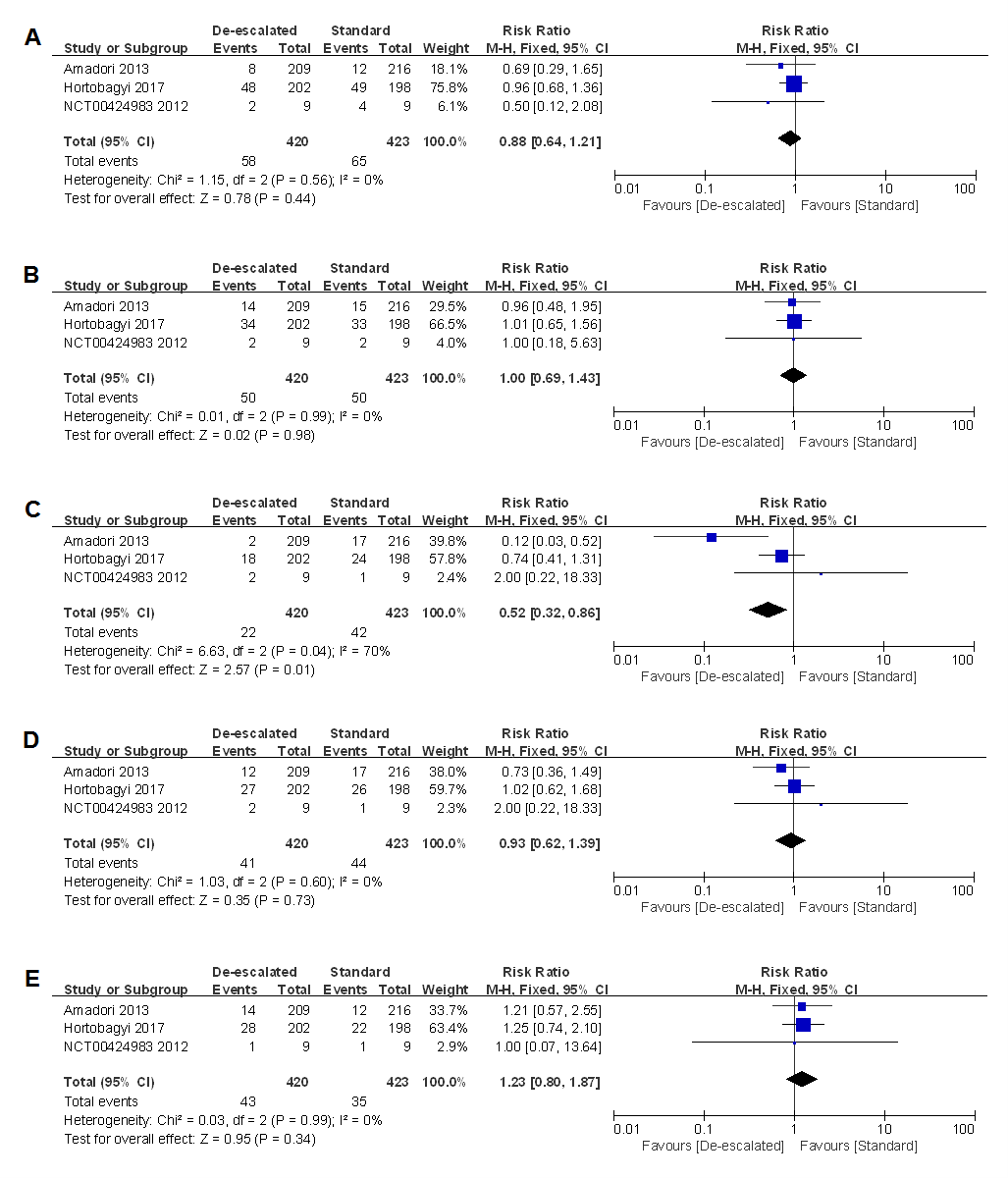


**Figure S 4 On-study (A) pain in extremity, (B) headache, (C) dizziness, (D) edema peripheral and (E) cough**


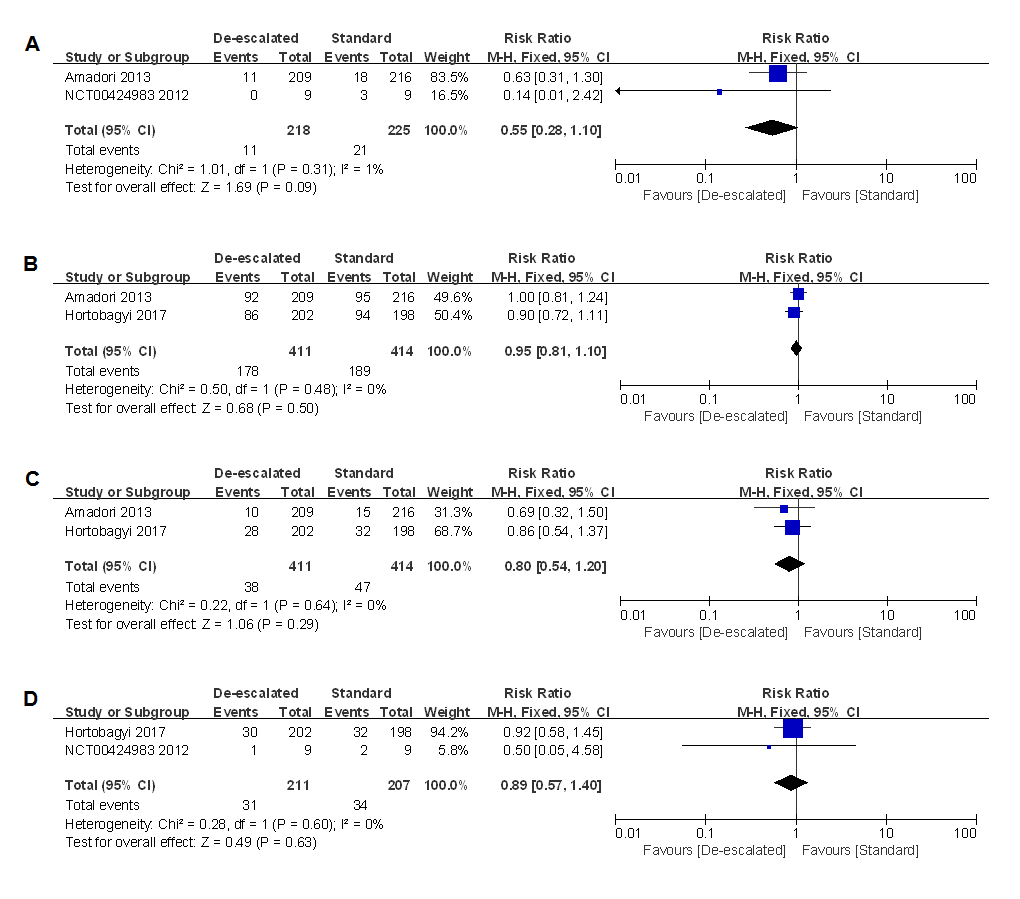


**Figure S 5 On study (A) neutropenia, (B) muculoskeletal pain, (C) dyspnoea and (D) grade 3 to 4 AEs**

**Table S 1 Individual types of SREs**

| Study | Drug | Radiation to bone | | Pathological fractures | | Surgery involving bone | | Spinal cord compression | |
| --- | --- | --- | --- | --- | --- | --- | --- | --- | --- |
|  |  | Q12w,  n/N | Q3-4w, n/N | Q12w,  n/N | Q3-4w, n/N | Q12w,  n/N | Q3-4w, n/N | Q12w,  n/N | Q3-4w, n/N |
| Hortobagyi | Zoledronate | 23/203 | 29/200 | / | / | / | / | / | / |
| Himelstein | Zoledronate | 163/911 | 185/911 | 79/911 | 62/911 | 42/911 | 22/911 | 30/911 | 23/911 |
| Amadori | Zoledronate | 22/209 | 24/216 | 10/209 | 9/216 | 2/209 | 1/216 | 2/209 | 1/216 |
| Coleman | Zoledronate | 2/19 | 2/19 | / | / | / | / | / | / |
| NCT00424983 | Zoledronate | 1/9 | 0/9 | 1/9 | 1/9 | 0/9 | 0/9 | 0/9 | 0/9 |
| Aggregate Totals | | 211/1351 | 240/1355 | 90/1129 | 72/1136 | 44/1129 | 23/1136 | 32/1129 | 24/1136 |
| Incidence Rates (%) | | 15.62 | 17.71 | 7.97 | 6.34 | 3.90 | 2.02 | 2.83 | 2.11 |
